# Body Mass Index and Head and Neck Cancer Risk

**DOI:** 10.2188/jea.JE20250218

**Published:** 2025-12-05

**Authors:** Shih-Wei Lai, Kuan-Fu Liao

**Affiliations:** 1 School of Medicine, China Medical University, Taichung, Taiwan; 2 Department of Family Medicine, China Medical University Hospital, Taichung, Taiwan; 3 Division of Hepatogastroenterology, Department of Internal Medicine, Taichung Tzu Chi Hospital, Taichung, Taiwan

Dear Editor,

We read with interest the recent article by Suzuki et al exploring the association between body mass index (BMI) and head and neck cancer (HNC) risk in a large Japanese cohort.^1^ The authors should be commended for their rigorous analysis and valuable contribution to cancer epidemiology, particularly in Asian populations. We would like to raise a few points for discussion with the authors and readers.

Given that smoking and alcohol consumption are both established risk factors for HNC,^2^^,^^3^ the study appropriately presents stratified analyses by these lifestyle factors. For example, the main finding is that low BMI (<18.5 kg/m^2^) is significantly associated with increased HNC risk in the overall population (hazard ratio [HR] 2.75; 95% confidence interval [CI], 1.63–4.64).^1^ This association was consistently observed across subgroups, including never-smokers (HR 2.65; 95% CI, 1.16–6.05), former/current smokers (HR 3.09; 95% CI, 1.54–6.20), non/occasional drinkers (HR 2.25; 95% CI, 1.04–4.89), and regular drinkers (HR 3.52; 95% CI, 1.53–8.09).^1^ Although the association between low BMI and increased HNC risk was consistently observed across smoking and alcohol consumption strata, the potential modifying effect of these lifestyle factors—particularly regarding high BMI—remains unclear. Formal interaction testing would help clarify whether the observed differences in the BMI–HNC association across smoking and alcohol consumption categories represent true effect modification or are simply due to chance. Incorporating interaction terms into multivariable Cox models is a standard and essential approach in epidemiologic research when stratified analyses suggest heterogeneity. Without such testing, it remains uncertain whether the subgroup differences represent true effect modification or are due to chance. We, therefore, encourage the authors to conduct and report formal interaction analyses, which would enhance the interpretability of their findings and provide stronger guidance for risk stratification and public health messaging.
